# Matrine Attenuates COX-2 and ICAM-1 Expressions in Human Lung Epithelial Cells and Prevents Acute Lung Injury in LPS-Induced Mice

**DOI:** 10.1155/2016/3630485

**Published:** 2016-01-05

**Authors:** Chian-Jiun Liou, You-Rong Lai, Ya-Ling Chen, Yi-Hsien Chang, Zih-Ying Li, Wen-Chung Huang

**Affiliations:** ^1^Department of Nursing, Chang Gung University of Science and Technology, No. 261 Wenhua 1st Road, Guishan District, Taoyuan City 33303, Taiwan; ^2^Chang Gung Memorial Hospital at Linkou, Guishan District, Taoyuan City 33303, Taiwan; ^3^Department of Nutrition and Health Sciences, Chang Gung University of Science and Technology, No. 261 Wenhua 1st Road, Guishan District, Taoyuan City 33303, Taiwan; ^4^Research Center for Industry of Human Ecology, Chang Gung University of Science and Technology, No. 261 Wenhua 1st Road, Guishan District, Taoyuan City 33303, Taiwan; ^5^Graduate Institute of Health Industry Technology, Chang Gung University of Science Taiwan and Technology, No. 261 Wenhua 1st Road, Guishan District, Taoyuan City 33303, Taiwan

## Abstract

Matrine is isolated from* Sophora flavescens* and shows anti-inflammatory effects in macrophages. Here we evaluated matrine's suppressive effects on cyclooxygenase 2 (COX-2) and intercellular adhesion molecule-1 (ICAM-1) expressions in lipopolysaccharide- (LPS-) stimulated human lung epithelial A549 cells. Additionally, BALB/c mice were given various matrine doses by intraperitoneal injection, and then lung injury was induced via intratracheal instillation of LPS. In LPS-stimulated A549 cells, matrine inhibited the productions of interleukin-8 (IL-8), monocyte chemotactic protein-1, and IL-6 and decreased COX-2 expression. Matrine treatment also decreased ICAM-1 protein expression and suppressed the adhesion of neutrophil-like cells to inflammatory A549 cells.* In vitro* results demonstrated that matrine significantly inhibited mitogen-activated protein kinase phosphorylation and decreased nuclear transcription factor kappa-B subunit p65 protein translocation into the nucleus.* In vivo* data indicated that matrine significantly inhibited neutrophil infiltration and suppressed productions of tumor necrosis factor-*α* and IL-6 in mouse bronchoalveolar lavage fluid and serum. Analysis of lung tissue showed that matrine decreased the gene expression of proinflammatory cytokines, chemokines, COX-2, and ICAM-1. Our findings suggest that matrine improved lung injury in mice and decreased the inflammatory response in human lung epithelial cells.

## 1. Introduction

Acute lung injury (ALI) is characterized by atelectasis of lung airspaces, reducing the total lung capacity [1]. In ALI cases, the lung tissue releases large amounts of inflammatory cytokines, chemokines, and inflammatory media, such as nitric oxide and cyclooxygenase 2 (COX-2) [2]. ALI also causes lung cells to secrete more proteases, leading to alveolar cell damage and fluid accumulation in alveoli, causing edema in the lung tissue [3]. Alveolar damage results in increased vascular permeability of blood vessels and neutrophil infiltration in the lung tissue, further exacerbating lung inflammation and pulmonary edema and potentially causing respiratory failure and death [4].

ALI commonly occurs as an early symptom of sepsis or bacterial infection of the respiratory tract [2]. Gram-negative bacterial invasion of the lungs can cause bacterial pneumonia and ALI [5]. Lipopolysaccharide (LPS) is a cell wall component of Gram-negative bacteria and can induce an innate immunity response, activating immune cells to combat microbial infection [6]. Thus, LPS is widely used to induce acute lung inflammation in animal models. Activated macrophages and lung epithelial cells reportedly release proinflammatory cytokines and chemokines, aggravating ALI progression. Prior studies have used lung epithelial cells to evaluate the anti-inflammatory responses of drugs or natural compounds during LPS-induced inflammatory response [7, 8].

In China and Taiwan, the root of* Sophora flavescens* (Leguminosae) is used to treat fever, jaundice, urinary tract infections, and pyogenic infections [9]. Matrine is isolated from* S. flavescens* and can reportedly attenuate cerebral ischemic injury in mice and induce apoptosis of chronic myeloid leukemia cells, osteosarcoma cells, and cholangiocarcinoma cells [9, 10]. Zhang et al. previously demonstrated that matrine suppressed proinflammatory cytokine production in LPS-stimulated mouse macrophages [11]. Previously, our group also found that matrine could improve eosinophil infiltration, airway hyperresponsiveness, and Th2-associated cytokine production in asthmatic mice [12]. However, the mechanisms of matrine's anti-inflammatory activity in lung epithelial cells are largely unclear.

In the present study, we investigated whether matrine reduced inflammatory responses and production of intercellular adhesion molecule-1 (ICAM-1) in lung epithelial cells. We also examined whether matrine protected and prevented acute lung injury in LPS-induced mice.

## 2. Materials and Methods

### 2.1. Matrine and Cell Culture

Matrine (≥99% by HPLC) was purchased from Sigma-Aldrich (St. Louis, MO, USA) and was dissolved in normal saline as previously described [12]. The human lung epithelial cell line A549 was purchased from the Bioresource Collection and Research Center (BCRC, Taiwan). A549 cells were cultured in DMEM medium (Invitrogen-Gibco, Paisley, Scotland) containing 2 mM glutamine, 100 U/mL penicillin and streptomycin, and 10% fetal bovine serum (Biological Industries, Haemek, Israel). Cells were incubated at 37°C in 5% CO_2_ humidified air and were subcultured twice each week.

### 2.2. Cell Viability Assay

Cell viability was assayed using 3-(4,5-dimethylthiazol-2-yl)-2,5-diphenyltetrazolium bromide (MTT; Sigma) as previously described [13]. In 96-well culture plates, A549 cells were treated with various matrine concentrations for 24 h. The plates were then washed, MTT solution was added, and the plates were incubated at 37°C for 4 h. Formazan crystals were dissolved in isopropanol, and cell viability was spectrophotometrically measured at 570 nm with a microplate reader (Multiskan FC; Thermo, Waltham, MA, USA).

### 2.3. Animals

Female BALB/c mice were purchased from the National Laboratory Animal Center in Taiwan. Mice were maintained in animal housing with a thermostat and central air conditioning. The care and housing of the mice were approved by the Laboratory Animal Care Committee of Chang Gung University of Science and Technology (IACUC approval number: 2014-007).

### 2.4. Acute Lung Injury and Drug Treatment

The mice were randomly divided into four groups of 10 mice each: normal control mice (N group), the LPS group, 10 mg/kg matrine plus LPS (M10 group), and 20 mg/kg matrine plus LPS (M20 group). On days 1–7 of the experiment, mice were intraperitoneally injected with matrine. On day 8, the mice were anesthetized with isoflurane (Aesica, Kent, UK) and received intratracheal injection of 50 *μ*L LPS (1 *μ*g/mL) or normal saline. Four hours after LPS administration, the mice were sacrificed and we collected samples of serum, bronchoalveolar lavage fluid (BALF), and lung tissue.

### 2.5. Bronchoalveolar Lavage Fluid and Cell Count

BALF was collected as described previously [14]. Briefly, the mouse trachea was intubated and the lungs were flushed with 1 mL normal saline. To determine the neutrophil cell count in BALF, the cells were stained with Liu stain solution (Polysciences, Inc., Taipei, Taiwan). The supernatants were also measured for cytokine and chemokine productions.

### 2.6. Histological Analysis of Lung Tissue

Lung tissues were fixed in 10% formalin, embedded in paraffin, and cut into 6 *μ*m sections. The slides were stained with hematoxylin and eosin (HE). The neutrophil infiltration assay was performed as described previously [15].

### 2.7. Serum Collection

Following anesthetization, serum was collected from the orbital vascular plexus. Serum samples were centrifuged at 6000 rpm for 5 min as previously described [16], followed by storage at −80°C until use.

### 2.8. ELISA for Cytokine and Chemokine Production

In an* in vitro* experiment, A549 cells were pretreated with matrine (100–400 *μ*M) for 1 h in 24-well plates and then they were stimulated with LPS (1 *μ*g/mL) and cultured for 24 h. We then used specific ELISA kits (R&D Systems, Minneapolis, MN, USA) to assay IL-8, IL-6, monocyte chemotactic protein-1 (MCP-1), CCL5, and ICAM-1 production. Optical density (OD) was spectrophotometrically measured at 450 nm using a microplate reader (Multiskan FC, Thermo). In our* in vivo* study, we also used ELISA to measure cytokines and chemokines in serum and BALF.

### 2.9. RNA Isolation and Real-Time PCR for Gene Expression

Total RNA was extracted from lung tissue using TRIzol reagent (Life Technologies, Carlsbad, CA, USA). From this RNA, we synthesized cDNA using a cDNA synthesis kit (Life Technologies). We then used a real-time PCR system kit (Bio-Rad Laboratories, Hercules, CA, USA) with a spectrofluorometric thermal cycler (iCycler; Bio-Rad) to detect cDNA gene expression.  Table 1 presents the specific gene primers that were used.

### 2.10. Cell-Cell Adhesion Assay

A549 cells were treated with matrine and incubated with LPS (1 *μ*g/mL) for 24 h. Then, neutrophil-like HL-60 cells were treated with calcein AM solution (Sigma) and cocultured with A549 cells for 1 h. We observed the adhesion of HL-60 cells to A549 using fluorescence microscopy (Olympus, Tokyo, Japan).

### 2.11. Preparation of Total and Nuclear Proteins

Following treatment with matrine for 1 h, A549 cells were stimulated with LPS (1 *μ*g/mL) for 30 min to detect protein phosphorylation or for 24 h to evaluate total protein expressions. Proteins were extracted using protein lysis buffer containing protease and phosphatase inhibitors (Sigma). Nuclear proteins were extracted using NE-PER nuclear and cytoplasmic extraction reagent kits (Pierce, Rockford, IL, USA). All proteins were quantified using the BCA protein assay kit (Pierce).

### 2.12. Western Immunoblot Analysis

Equal amounts of protein were separated on 10% SDS polyacrylamide gels and then transferred to polyvinylidene fluoride (PVDF) membranes (Millipore, Billerica, MA, USA). The PVDF membranes were incubated overnight at 4°C with primary antibodies, including COX-2, I*κ*B-*α*, phosphorylated-I*κ*B-*α*, lamin B1, p65, phosphorylated-I*κ*B-*α* (Santa Cruz, CA, USA), ERK1/2, p38, JNK, phosphorylated-ERK1/2, phosphorylated-p38, phosphorylated-JNK (Millipore), ICAM-1, and *β*-actin (Sigma). After the overnight incubation, the membrane was washed with TBST buffer (150 mM NaCl; 10 mM Tris, pH 8.0; and 0.1% Tween 20) and incubated with HRP-conjugated secondary antibodies for 1 h. Finally, the membranes were washed and processed with Luminol/Enhancer Solution (Millipore) for detection and quantification of the specific protein using the BioSpectrum 600 system (UVP, Upland, CA, USA).

### 2.13. Transfection and Luciferase Assays

Transfected pNF*κ*B-Luc plasmid evaluated NF-*κ*B activity as previously described [13]. Briefly, A549 cells were transfected with pNF*κ*B-Luc plasmid (Stratagene, California, USA) and cells were pretreated with matrine for 1 h and were stimulated with LPS for 4 h. The luciferase activity was assayed using a Multi-Mode Microplate Reader (BioTek Synergy HT, Bedfordshire, United Kingdom).

### 2.14. Statistical Analysis

All data were analyzed using one-way analysis of variance (ANOVA), followed by Tukey-Kramer method for multiple comparisons. Values are expressed as mean ± SEM. A *P* value of <0.05 was considered to indicate a statistically significant difference.

## 3. Results

### 3.1. Effects of Matrine on Inflammatory Response in Activated A549 Cells

**Figure 1:**
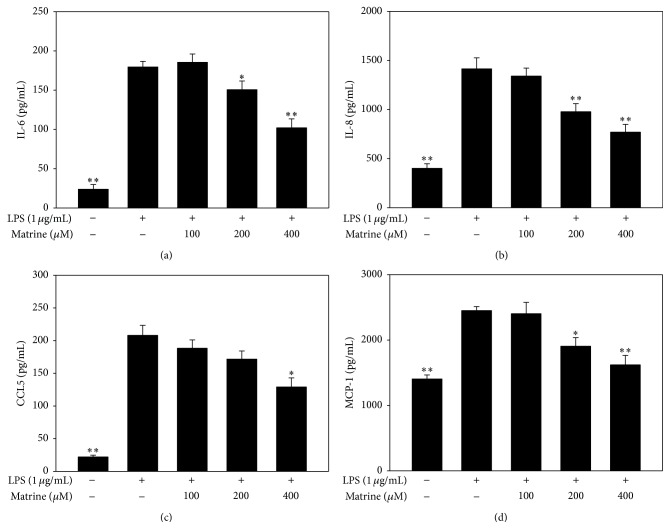
Matrine affects cytokine and chemokine productions in LPS-stimulated A549 cells. ELISA was used to measure the levels of IL-6 (a), IL-8 (b), CCL5 (c), and MCP-1 (d). All data are presented as mean ± SEM. ^*∗*^ *P* < 0.05 compared with the LPS control group. ^*∗∗*^ *P* < 0.01 compared with the LPS control group.

At doses of ≤400 *μ*M, matrine did not affect the viability of A549 cells (data not shown). Thus, matrine concentrations of 100–400 *μ*M were used in the* in vitro* experiments. First, we investigated how matrine modulated cytokine and chemokine production in LPS-stimulated A549 cells (Figure 1). We found that matrine significantly decreased IL-6 production in a concentration-dependent manner: IL-6 production with LPS alone, 179.4 ± 7.9 pg/mL; with 100 *μ*M matrine, 185.4 ± 10.2 pg/mL (*P* = 0.28 versus LPS alone); 200 *μ*M matrine, 150.5 ± 11.2 pg/mL (*P* < 0.05 versus LPS alone); 400 *μ*M matrine, 101.9 ± 11.5 pg/mL (*P* < 0.01 versus LPS alone). Matrine also dose-dependently decreased the levels of IL-8, MCP-1, and CCL5 in LPS-stimulated A549 cells.

### 3.2. Effects of Matrine on LPS-Induced COX-2 and ICAM-1 Protein Expressions

**Figure 2:**
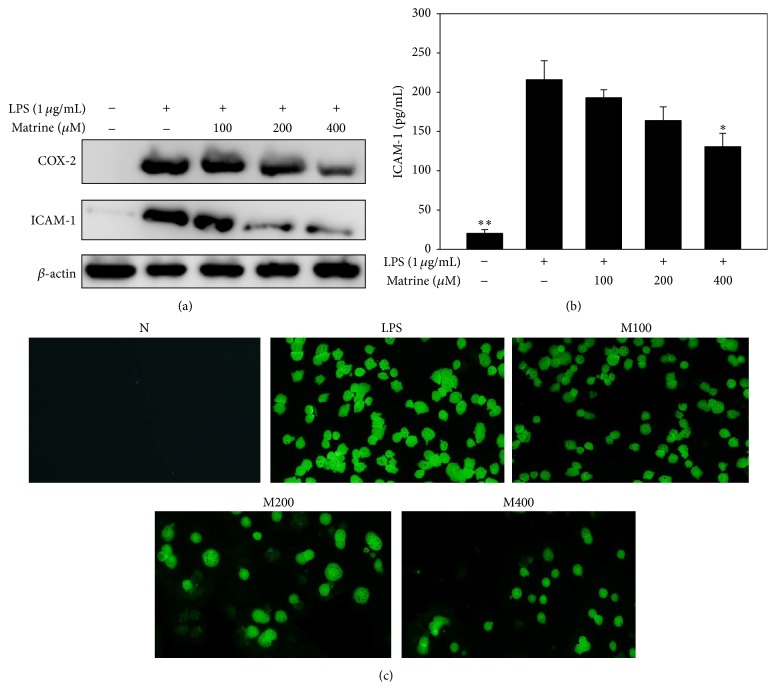
Matrine affects the LPS-induced productions of COX and ICAM-1 in A549 cells. COX-2 and ICAM-1 protein levels were detected using Western blots with *β*-actin expression as an internal control (a), and ICAM-1 levels were measured by ELISA (b). (c) Treatment with 100–400 *μ*M matrine (M) inhibited the adherence of neutrophil-like HL-60 cells to active A549 cells. Data are presented as the mean ± SEM. ^*∗*^ *P* < 0.05 compared to LPS alone.

A549 cells were pretreated with matrine, followed by stimulation with LPS for 24 h. Matrine pretreatment significantly suppressed COX-2 protein expression in a concentration-dependent manner compared with LPS-stimulated A549 cells (Figure 2(a)). ELISA (Figure 2(b)) and Western blot analysis (Figure 2(a)) also showed that matrine significantly inhibited ICAM-1 expression: LPS alone, 215.9 ± 24.3 pg/mL; matrine 100 *μ*M, 192.9 ± 10.3 pg/mL (*P* = 0.12 versus LPS alone); matrine 200 *μ*M, 163.8 ± 18.4 pg/mL (*P* < 0.05 versus LPS alone); matrine 400 *μ*M, 130.6 ± 17.5 pg/mL (*P* < 0.01 versus LPS alone). Inflammatory lung epithelial cells express ICAM-1 to promote neutrophil adhesion [17]. Correspondingly, we also found that matrine treatment decreased the adherence of HL-60 cells to LPS-stimulated A549 cells (Figure 2(c)).

### 3.3. Effects of Matrine on LPS-Induced NF-*κ*B Activation and Phosphorylation of MAPK Pathways in Human Lung Epithelial Cells

**Figure 3:**
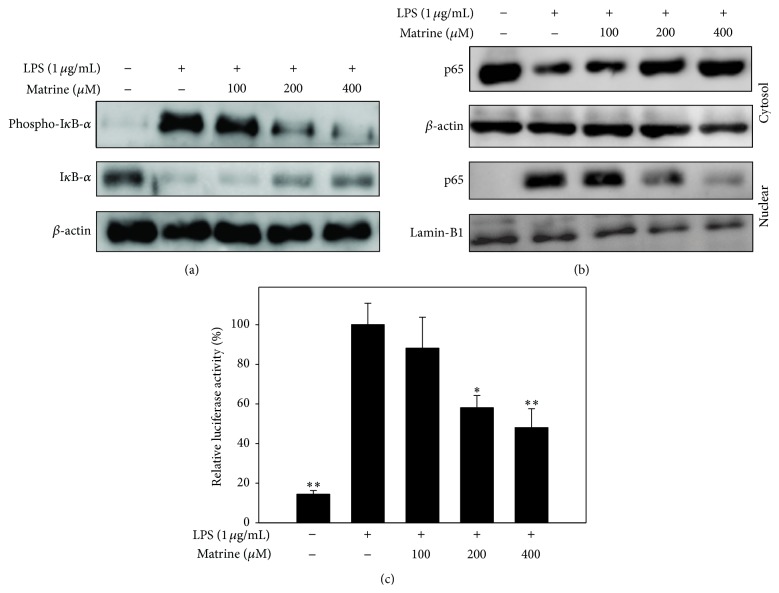
Matrine inhibits the nuclear translocation of NF-*κ*B in A549 cells. (a) Effects of matrine on the LPS-induced phosphorylation of I*κ*B-*α*, with total I*κ*B-*α* levels used as internal controls. (b) To assess the nuclear translocation of NF-*κ*B, cells were pretreated with different matrine concentrations for 1 h and then incubated with LPS (1 *μ*g/mL) for 1 h. The internal controls were lamin B1 in the nucleus and *β*-actin in the cytosol. (c) Matrine suppressed luciferase activity by the NF-*κ*B promoter assay. Three independent experiments were analyzed and compared with the LPS-treated control group. Data are presented as the mean ± SEM. ^*∗*^ *P* < 0.05 compared to LPS alone.

**Figure 4:**
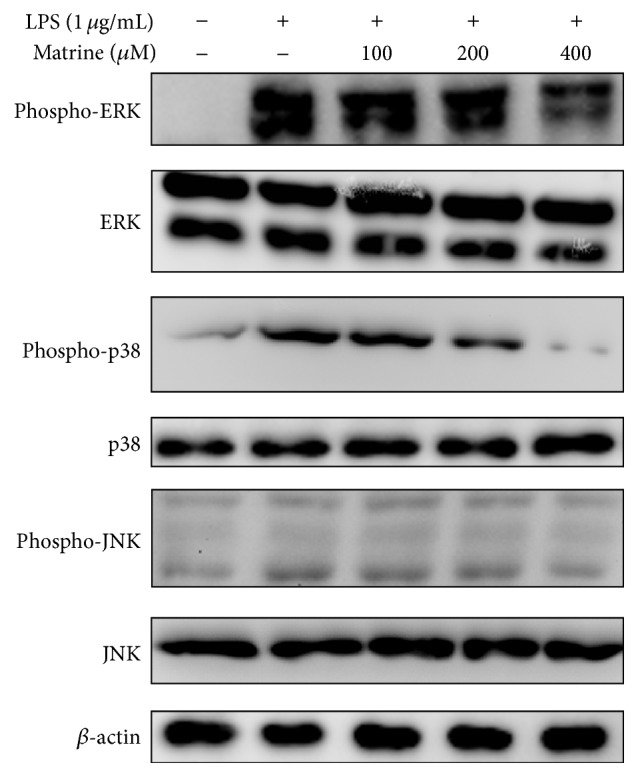
Matrine affects LPS-induced phosphorylation of MAPK pathway molecules. Western blots showed the effects of the indicated matrine concentrations on the phosphorylation of ERK (*top two rows*), p38 (*middle two rows*), and JNK (*bottom two rows*). Three independent experiments were analyzed and compared with the LPS-treated group.

Upon finding that matrine decreased productions of chemokines, proinflammatory cytokines, and COX-2 in LPS-stimulated A549 cells, we further investigated whether matrine attenuated activation of the NF-*κ*B and MAPK pathways in these cells. In unstimulated cells, I*κ*B holds the NF-*κ*B complex (p65 and p50) in the cytoplasm, while LPS stimulation induces phosphorylation of I*κ*B-*α*, increasing the release of p65 into the nucleus [18]. Here we found that matrine significantly inhibited I*κ*B-*α* phosphorylation and decreased I*κ*B-*α* degradation, thus attenuating p65 translocation into the nucleus compared with that observed in LPS-stimulated A549 cells (Figures 3(a) and 3(b)). NF-*κ*B activity was evaluated using a promoter luciferase assay, and it was found that matrine could significantly decrease luciferase activity (Figure 3(c)). We also observed that matrine suppressed phosphorylation of ERK1/2, JNK, and p38 compared with LPS-stimulated A549 cells (Figure 4).

### 3.4. Matrine Inhibits Neutrophil Infiltration of LPS-Induced Lung Injury in Mice

**Figure 5:**
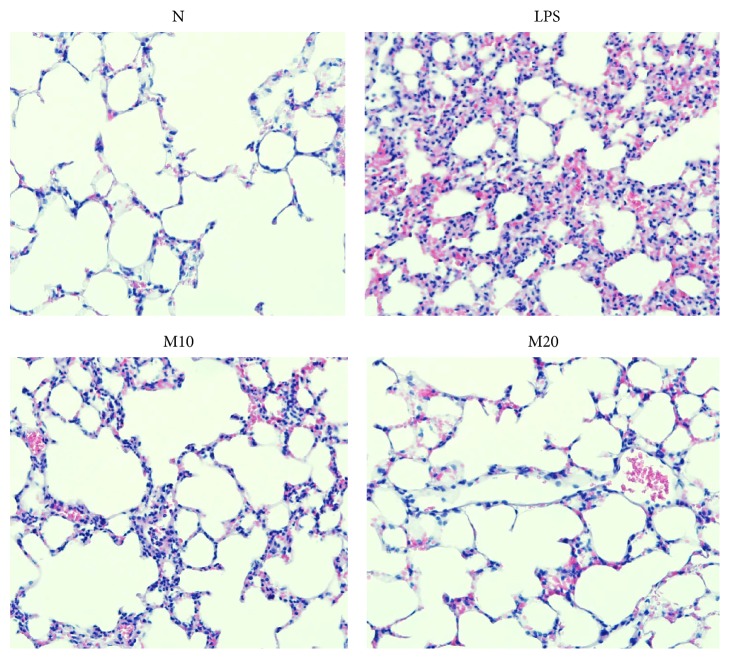
Matrine inhibits neutrophil infiltration. Hematoxylin and eosin staining was used for histological examination of airway inflammation in lung tissue (magnification, 400x). N group: normal control mice; LPS group: LPS treatment only; M10 group: 10 mg/kg matrine plus LPS; M20 group: 20 mg/kg matrine plus LPS.

After the sacrifice of mice, lung tissue was fixed with formalin and HE stained to analyze the distribution of neutrophils in lung sections. The LPS group showed greater neutrophil infiltration compared to the N group (Figure 5). Moreover, matrine treatment significantly suppressed neutrophil infiltration in the lung biopsy sections of mice.

### 3.5. Matrine Reduced the Neutrophil Count in BALF from LPS-Challenged Mice

**Figure 6:**
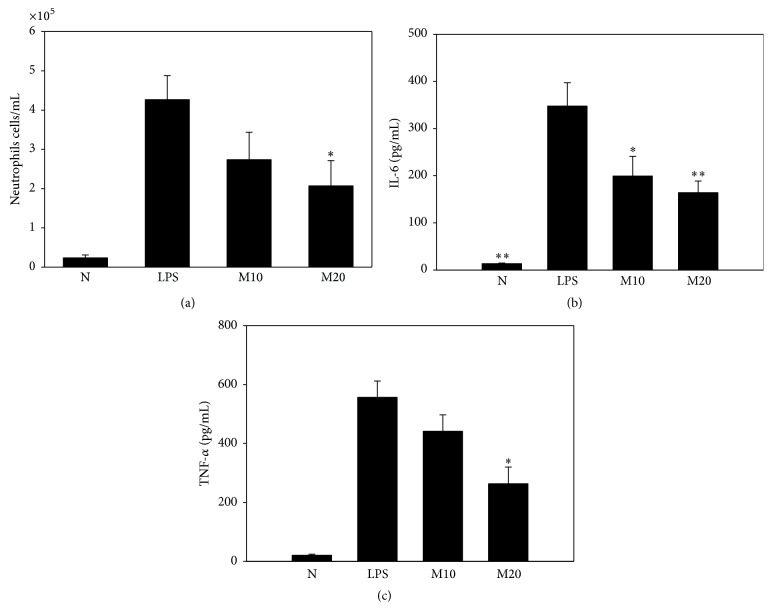
Matrine affects neutrophil counts and cytokine and chemokine levels in BALF. In the different treatment groups, we analyzed neutrophil cell counts (a), and the levels of IL-6 (b) and TNF-*α* (c) were measured by ELISA. N group: normal control mice; LPS group: LPS treatment only; M10 group: 10 mg/kg matrine plus LPS; M20 group: 20 mg/kg matrine plus LPS. All data are presented as mean ± SEM. ^*∗*^ *P* < 0.05 compared with the LPS control group. ^*∗∗*^ *P* < 0.01 compared with the LPS control group.

Evaluation of inflammatory cells in BALF revealed that matrine suppressed neutrophil infiltration in acute lung injury mice. Intraperitoneal injection of matrine in LPS-induced acute lung injury mice led to a significant decrease of neutrophils compared with the LPS group: LPS alone, 4.3 × 10^5^ ± 6.1 × 10^4^ neutrophils/mL; M10, 2.7 × 10^5^ ± 7.1 × 10^4^ neutrophils/mL (*P* = 0.08 versus LPS alone); M20, 2.0 × 10^5^ ± 6.1 × 10^4^ neutrophils/mL (*P* < 0.05 versus LPS alone) (Figure 6(a)).

### 3.6. Effects of Matrine on Cytokine and Chemokine Levels in BALF

**Figure 7:**
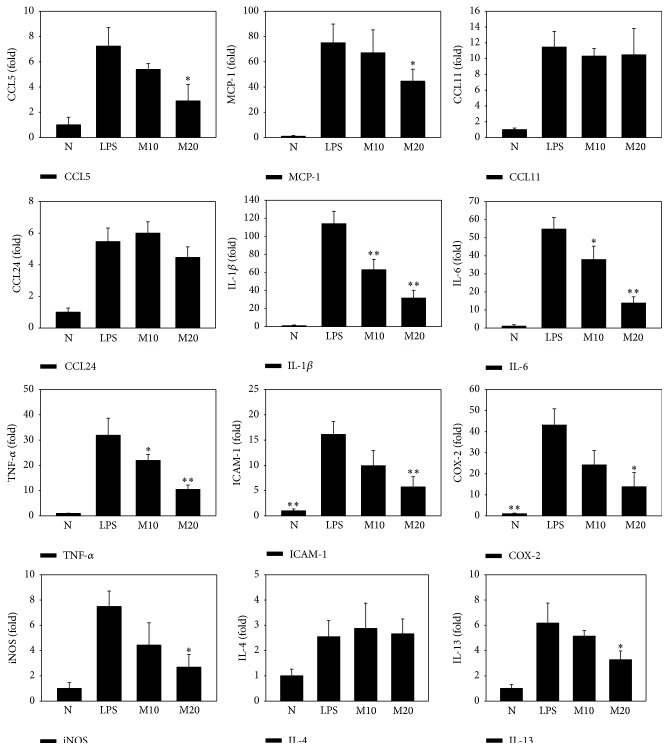
Matrine affects the expressions of cytokines, chemokines, and inflammatory mediators in the lung. Gene expressions were determined using real-time RT-PCR. Fold expression levels were measured relative to *β*-actin expression (internal control). Data are presented as mean ± SEM. ^*∗*^ *P* < 0.05 compared with LPS control mice. ^*∗∗*^ *P* < 0.01 compared with LPS control mice.

Acute lung injury mice treated with matrine exhibited lower IL-6 levels: LPS alone, 347.8 ± 97.6 pg/mL; M10, 199.3 ± 8.3 pg/mL (*P* < 0.05 versus LPS alone); M20, 163.9 ± 49.8 pg/mL (*P* < 0.01 versus LPS alone) (Figure 6(b)). Matrine treatment also reduced TNF-*α* levels in these mice: LPS alone, 556.3 ± 111.9 pg/mL; M10, 441.0 ± 71.1 pg/mL (*P* = 0.11 versus LPS alone); M20, 262.9 ± 69.1 pg/mL (*P* < 0.05 versus LPS alone) (Figure 6(c)). Using real-time PCR to evaluate gene expression, we found that matrine significantly decreased levels of IL-1*β*, IL-6, TNF-*α*, IL-13, MCP-1, and CCL5 in lung injury tissue (Figure 7). However, matrine did not suppress the gene expressions of IL-4, CCL11, or CCL24. We further found that matrine inhibited the gene expressions of ICAM-1, iNOS, and COX-2 in lung injury tissue.

### 3.7. Effects of Matrine on Phosphorylation of NF-*κ*B and MAPK Pathways in Lung Injury Tissue

**Figure 8:**
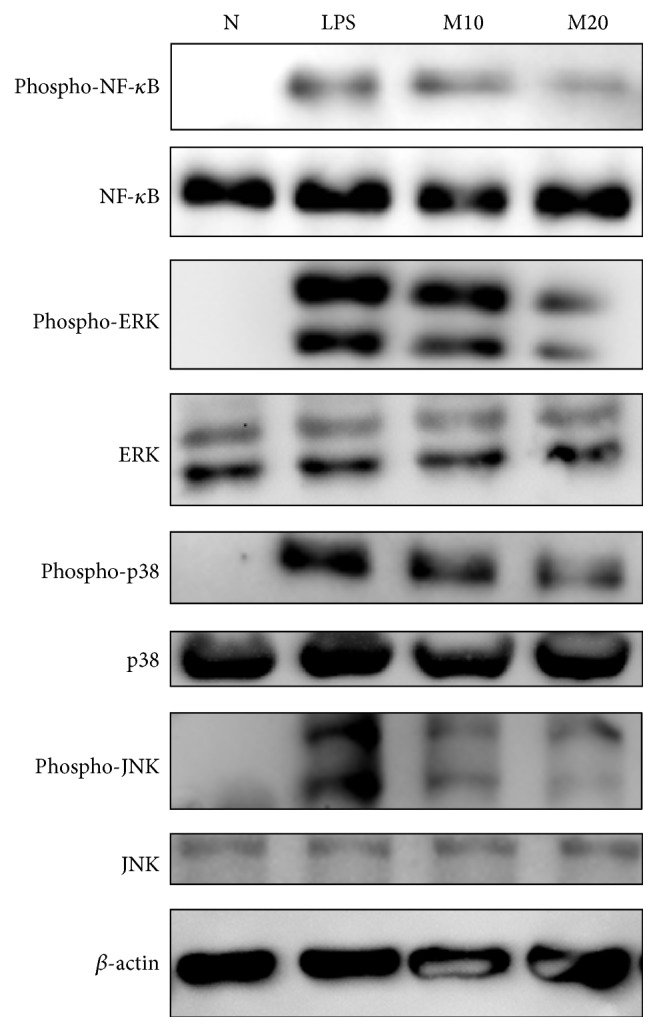
Matrine affects the LPS-induced nuclear phosphorylation of NF-*κ*B and phosphorylation of MAPK pathway molecules. Phosphorylation in lung tissue was analyzed by Western blots. Three independent experiments were analyzed and results were compared with the LPS-treated group.

Matrine significantly inhibited phosphorylation of p65 compared with that in LPS-stimulated lung injury tissue. Matrine also suppressed phosphorylation of ERK1/2, JNK, and p38 in the lung injury tissue (Figure 8).

### 3.8. Effects of Matrine on Cytokine Production in Serum

**Figure 9:**
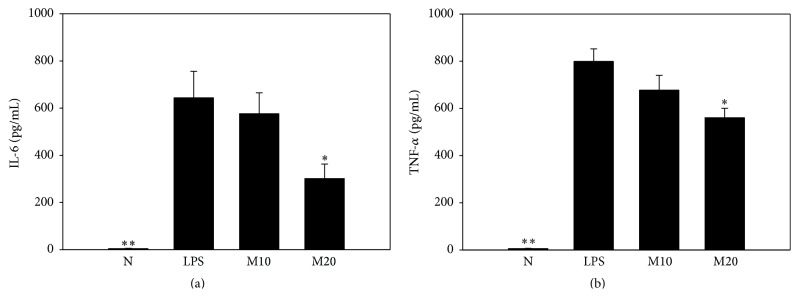
Matrine affects the serum levels of inflammatory cytokines, including IL-6 (a) and TNF-*α* (b). All data are presented as means ± SEM. ^*∗*^ *P* < 0.05 compared with the LPS control group.

Serum analysis revealed significantly reduced IL-6 production and TNF-*α* production in the M20 group compared to in the LPS group (Figure 9).

## 4. Discussion

Matrine is a major active component isolated from* Sophora flavescens* (Kushen), which reportedly shows many biological and medical properties, including improved inflammatory responses of the central nervous system, antitumor activities, and immunity-regulating effects [10, 19]. We previously demonstrated that matrine suppresses eosinophil infiltration in lung tissue and improves airway hyperresponsiveness and suppresses tracheal goblet cell hyperplasia in asthmatic mice [12]. Moreover, Zhang et al. described lung histopathological changes and inflammatory response at 18 h after LPS injection in mice, reporting that matrine decreased myeloperoxidase and malondialdehyde activities and inhibited reactive oxygen species and oxidative stress in BALF [20].

In our present study, we investigated whether matrine improved the inflammatory response in acute lung injury mice at 4 h after LPS injection into the trachea and also examined inflammatory responses in LPS-stimulated human lung epithelial cells. Our results showed that matrine significantly suppressed COX-2 expression and productions of IL-6, IL-8, MCP-1, and CCL5 in LPS-stimulated A549 cells. Matrine treatment also suppressed ICAM-1 expressions and, correspondingly, reduced LPS-stimulated A549 cell adherence to neutrophils. Finally, matrine suppressed components of inflammatory signaling pathways, including the NF-*κ*B and MAPK pathways.

LPS is an important component of Gram-negative bacteria cell walls [21]. Upon infection with Gram-negative bacteria, LPS release leads to inflammation and fever symptoms, potentially causing severe acute or chronic organ congestion, edema, or even septic shock and death [22]. LPS stimulates I*κ*B phosphorylation, emancipating NF-*κ*B (containing p50 and p65) to translocate into the nucleus. In the nucleus, p65 binds the promoters of inflammatory genes, leading to the expressions of proinflammatory cytokines, chemokines, COX-2, and ICAM-1 in LPS-stimulated A549 cells [21, 23]. MAPK pathways also modulate NF-*κ*B activation to alter COX-2 and ICAM-1 expressions [24]. Our present experiments demonstrated that matrine significantly inhibited the secretion of proinflammatory cytokines and chemokines from LPS-induced A549 lung epithelial cells. This effect may have been because matrine inhibited MAPK and NF-*κ*B activation. These results suggest that matrine treatment could improve the inflammatory response in cases of lung bacterial infections.

Previously, Zhang et al. investigated the administration of matrine after 30 min of LPS stimulation and reported that matrine reduces lung inflammation and oxidation by inhibiting inflammatory signal pathways [20]. However, their protocol involved LPS injected intravenously after 60 hours, and the survival rate was only 20%. Mice given 20 mg/kg matrine did not show an increased survival rate; mice given 100 mg/kg matrine showed a 60% survival rate and decreased lung damage [20]. In contrast, our present experimental model of lung injury involved intratracheal LPS injection to induce local inflammatory lung injury, which seems to be more suitable for assessing the inflammatory response. Furthermore, our experiment mainly investigated whether matrine could provide protection against lung injury in mice. The mice were treated with intraperitoneal matrine injection for continuous 7 days, followed by intratracheal LPS injection on the 8th day, and the mice were sacrificed four hours later. The experimental process did not lead to the death of any mice, and our model is more appropriate for observing the early response of acute lung injury and for evaluating the protective effects of matrine in early lung injury. Our results showed that 20 mg/kg matrine could protect and prevent lung injury symptoms in this experimental model. Despite the differences between our animal experimental model and the lung injury mouse model used by Zhang et al. [20], both sets of experimental results suggest that matrine can reduce lung injury in mice.

In cases of bacterial infection, neutrophils in the lung can ingest invading bacteria and release large amounts of inflammatory mediators to induce an inflammatory response in the lung [25]. Our experiments showed that treatment with 20 mg/kg of matrine suppressed neutrophil infiltration in the lung and correspondingly reduced the number of neutrophils in BALF. Chemokines—particularly IL-8, MCP-1, and CCL5—induce neutrophil migration into inflammatory tissue [26]. Matrine inhibited the productions of IL-8, MCP-1, and CCL5 in LPS-stimulated A549 cells and suppressed MCP-1 and CCL5 gene expressions in mouse lung tissue. Inflammatory lung epithelial cells also express ICAM-1 adhesion molecules to promote neutrophil adsorption [18, 27]. Our experiments found that matrine suppressed ICAM-1 expression both* in vitro* and* in vivo*. Overall, our results suggest that matrine may be useful for reducing neutrophil infiltration in the inflammatory response of the lungs. This would be particularly useful, since lung inflammation and lung damage in LPS-induced acute lung injury are mainly caused by neutrophil infiltration. On the other hand, matrine did not effectively reduce the gene expressions of CCL11 and CCL24 in the lung and, therefore, matrine did not improve eosinophilic infiltration in this LPS-induced lung injury mouse model. Matrine also did not effectively suppress the gene expressions of the Th2-associated cytokines IL-4 and IL-13 in lung tissue.

## 5. Conclusion

In conclusion, our results demonstrate that matrine significantly suppressed the inflammatory response in human lung epithelial cells and blocked ICAM-1 expression, preventing acute lung injury in LPS-induced mice.

## Figures and Tables

**Table 1:** Primers used in real-time PCR analyses of mRNA expression.

Gene	Primers	(5′-3′ sequence)	GenBank accession number	Product size (bp)
IL-1*β*	Forward Reverse	CACTACAGGCTCCGAGATGA CGTTGCTTGGTTCTCCTTGT	NM_008361	143

IL-4	Forward Reverse	TCCGTGCTTGAAGAAGAACTC GTGATGTGGACTTGGACTCATT	NM_021283.2	116

IL-6	Forward Reverse	AGGACCAAGACCATCCAATTCA GCTTAGGCATAACGCACTAGG	NM_031168.1	97

IL-13	Forward Reverse	GCTCCAGCATTGAAGCAGTG CGTGGCAGACAGGAGTGTT	NM_008355.3	141

TNF-*α*	Forward Reverse	GCACCACCATCAAGGACTC AGGCAACCTGACCACTCTC	NM_013693	96

MCP-1	Forward Reverse	TTCCACAACCACCTCAAGCA TTAAGGCATCACAGTCCGAGTC	NM_011333	80

CCL5	Forward Reverse	CGAAGGAACCGCCAAGTGT AGGACTAGAGCAAGCAATGAC	NM_013653.3	139

CCL11	Forward Reverse	GGCTTCATGTAGTTCCAGAT CCATTGTGTTCCTCAATAATCC	NM_011330.3	145

CCL24	Forward Reverse	AGGCAGTGAGAACCAAGT GCGTCAATACCTATGTCCAA	NM_019577.4	102

iNOS	Forward Reverse	TTCCACAACCACCTCAAGCA TTAAGGCATCACAGTCCGAGTC	NM_010927	83

COX-2	Forward Reverse	ACCAGCAGTTCCAGTATCAGA CAGGAGGATGGAGTTGTTGTAG	NM_011198	143

ICAM-1	Forward Reverse	AACAGAATGGTAGACAGCAT TCCACCGAGTCCTCTTAG	NM_010493.2	113

*β*-actin	Forward Reverse	AAGACCTCTATGCCAACACAGT AGCCAGAGCAGTAATCTCCTTC	NM_007393.3	92
